# Recall bias affects pain and depression assessment in multimorbidity: a pilot study

**DOI:** 10.1017/S1463423626101406

**Published:** 2026-07-03

**Authors:** Irina Mindlis, M. Carrington Reid, Thomas L. Rodebaugh

**Affiliations:** 1 Parker Health Group Division of Geriatrics, Department of Family Medicine and Community Health, Robert Wood Johnson Medical School of Rutgers University, USA; 2 Division of Geriatrics and Palliative Medicine, Weill Cornell Medicine, USA; 3 Department of Psychology and Neuroscience, The University of North Carolina at Chapel Hill, USA

**Keywords:** depression, ecological momentary assessment, multimorbidity, older adults, recall bias

## Abstract

**Objectives:**

Older adults with multimorbidity (OAMM) commonly receive depression and pain management through primary care, where symptoms are typically assessed through retrospective recall. Even with validated questionnaires, recall bias has been reported in younger populations for both depression and pain. However, recall bias for depression and pain has not been explored in OAMM. We examined discrepancies between prospectively measured and recalled symptoms of depression and pain among OAMM.

**Methods:**

We analysed data generated from a 14-day pilot feasibility trial of ecological momentary assessment (EMA) in OAMM (N = 18, age range 67–95). We examined discrepancies between retrospectively assessed depression and pain intensity over two weeks compared to follow-up, baseline characteristics correlated with discrepancies, and recency effects.

**Results:**

We found overreporting across most symptoms, with the largest discrepancies between prospectively recorded and recalled symptom scores for pain intensity and fatigue (d = .49). There was no association between recalled and EMA-measured items for appetite and trouble sleeping. Pain intensity at day 14 was associated with discrepancies in recalled pain (r = −.52, p =.029), and both day 14 mood and negative self-thoughts were associated with discrepancies in trouble concentrating recall (r = −.67, p =.002 and p = −.72, p <.001, respectively) – suggesting recency effects.

**Conclusions:**

We found preliminary evidence of recall bias among OAMM, including overreporting and recency effects of pain and depression symptoms. Given the reliance on recall during primary care visits, more research is needed.

## Introduction

Pain and depression occur commonly in older adults with multimorbidity (OAMM; Scherer *et al*., 2016; Mcqueenie *et al*., 2021; Ronaldson *et al*., 2021). In primary care, screening for and managing these conditions among affected individuals typically consists of assessing patients’ symptoms at the time of scheduled visits, which occur several times a year for this expanding population. These episodic assessments often rely on retrospective, unstructured symptom inquiry or validated questionnaires (Mindlis *et al*., 2025). Retrospective assessments (unstructured or structured) are subject to recall bias (Henry *et al*., 1994; Urban *et al*., 2018; Horwitz *et al*., 2023) and can produce poor quality data (Horwitz *et al.*, 2023) as the most intense and proximal aspects of an experience can influence memory for depressive symptoms (Horwitz *et al*., 2023). Thus, patients’ reports during primary care visits may not accurately reflect actual depression or pain trajectories – leading to risk of over-and undertreatment such as medication titration and clinical inertia.

Recent studies have examined recall bias in retrospective symptom assessment through ecological momentary assessment (EMA). For example, in a study of recall bias of knee osteoarthritis symptoms, higher pain was reported at follow-up compared to prospective daily assessments (Previtali *et al*., 2022). Similarly, retrospective overreporting was found for gastrointestinal symptoms in patients with functional bowel disorders, when comparing prospective and retrospective assessments (Jones *et al*., 2022). However, recall bias in depressive and pain symptoms has not, to the authors’ knowledge, been examined previously in OAMM.

Therefore, we analysed data generated in a pilot trial evaluating the feasibility and acceptability of EMA among OAMM, with the goal of ascertaining the extent of discrepancies between recalled (retrospective) and daily (prospective) symptom reports. We also explored sociodemographic and clinical characteristics associated with greater symptom recall discrepancies between retrospective and prospective assessments.

## Methods

Participants were recruited through the geriatric primary care practice of Weill Cornell Medicine (3/2024–9/2024) if they were ≥60 years, had a depression diagnosis (chart documented or self-reported), **≥** two chronic illnesses, a smartphone, and were English-speaking. We excluded patients with dementia, bipolar disorder, and severe mental illness based on chart review and self-report. Full details of the study have been described elsewhere (Mindlis et al., 2026). Briefly, following informed consent, participants completed baseline validated questionnaires, followed by EMA surveys three times daily for two weeks. Within one week of completing all EMA surveys, participants completed a follow-up interview.

## Measures

### Depressive symptoms

Participants completed the Patient Health Questionnaire (PHQ-8) at baseline and follow-up. Scoring is identical to the PHQ-9 (Kroenke *et al*., 2001).

### Pain intensity

The PROMIS Pain Intensity 3a (Cella *et al*., 2010) scale assessed pain intensity over the past week at baseline and follow-up. Participants rated their pain at its *worst*, on *average*, and *currently* on a 5-point scale ranging from ‘had no pain’ to ‘very severe’.

### Sociodemographic characteristics

We assessed age, gender, race, ethnicity, education, income, and relationship status.

### EMA measures

For this analysis, we focused on EMA items assessing symptoms of depression and pain intensity in participants who completed all interviews.

### Depressive symptoms

The EMA survey included selected items from the PHQ-9 that might fluctuate in a two–three hour period and items assessing sleep and appetite (assessed once a day in the morning and evening surveys, respectively). One item assessing suicidal thoughts was excluded to maximize survey acceptability. Thus, participants rated PHQ-9 items for depressed mood, anhedonia, negative self-thoughts, fatigue, and trouble concentrating using wording adapted for brevity in previous EMA studies (Schleider, 2021), and given a time frame of ‘since my last survey’. The EMA survey also included a pain intensity item (‘What is your level of pain right now?’) (Cella *et al.*, 2010). Response options for all items ranged from 0 (Terrible) to 100 (Excellent) for sleep and appetite items, and from 0 (Not at all) to 100 (Extreme) for all other symptoms.

### Statistical analysis

Analysis was carried out using SPSS V 29. Average scores for EMA items were calculated. Given the discrepancy between the EMA items range (0–100) and the follow-up questionnaires (0–3 for PHQ items; 1–5 for pain intensity), the mean scores on EMA items were transformed to a 0–3 scale for depression symptoms, and a 1–5 scale for pain intensity. Specifically, pain intensity scores were partitioned into five categories: 0–20 (1), 21–40 (2), 41–60 (3), 61–80 (4), 81–100 (5); whereas depression symptom scores were partitioned into four categories: 0–25 (0), 26–50 (1), 51–75 (2), 76–100 (3). Trouble sleeping and appetite items were reverse coded so that higher scores indicated more difficulty sleeping and worse appetite. Discrepancy scores were calculated as the recall (score on the retrospective follow-up interview) minus the average EMA-measured prospective scores for the two weeks for all depressive symptoms, and for the past week for pain intensity. Associations between discrepancy scores and baseline characteristics were evaluated through correlations. Following previous approaches to recall bias (Jones *et al.*, 2022), we explored recency effects by correlating day 14 EMA scores with discrepancy recall scores.

## Results

Participants (*N* = 18) had an average age of 80.3 (*SD* = 7.8, range 67–95), were mostly female (89%) and non-Hispanic/Latino White (83%). Participants had significant depressive symptoms (mean PHQ-8 score of 8.7, *SD* = 5.8), including 39% who scored in the moderate-to-severe depression range. Over half (61%) reported moderate-to-severe pain intensity (i.e., score of 3 or greater on 1–5 scale of the PROMIS Pain Intensity 3a scale) at baseline.

**Figure 1.**
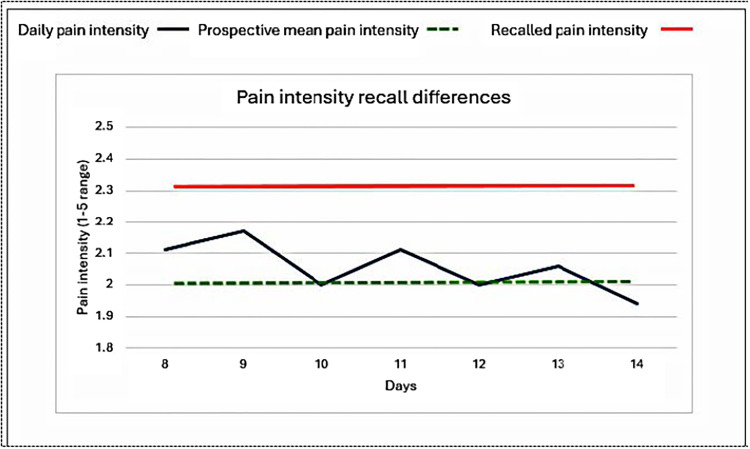
Representation of differences between daily and averaged prospectively measured pain intensity compared to recalled scores over one week.

Recall of depressive symptoms and pain intensity during the two-week study period was overall higher than the average EMA scores (See Table 1). The difference was small overall (average *d* = .18), ranging from the smallest for negative self-thoughts (*d* = .03) to the largest for pain intensity (See Figure 1) and fatigue (*d* = .49). EMA reports and recalled scores at follow-up correlated for all domains except appetite and trouble concentrating.

**Table 1.** Discrepancy between EMA-measured and recalled symptoms of depression and pain intensity during the two-week study period

Symptom	Mean difference	Difference *SD*	*d*	*r* ^1^
Pain intensity	.33	.69	.49	.800**
Depressed mood	.06	.76	.07	.632**
Anhedonia	.25	.79	.32	.691**
Sleep difficulties	.25	1.66	.15	.658**
Fatigue	.42	.84	.49	.552*
Appetite	.36	1.74	.21	.232
Negative thoughts of self	.03	.92	.03	.678**
Trouble concentrating	−.36	1.10	−.33	.390

*Note*: **p <.01, *p <.05. ^1^Represents the correlation between EMA-measured and recalled symptoms. Scores range from 1–5 for pain intensity, and 0–3 for PHQ-9 items.

We examined the associations between baseline characteristics and discrepancy scores. Greater pain intensity at baseline was associated with greater recall discrepancy (i.e., between retrospective and prospective assessments) for trouble sleeping (*r* = .60), but lower discrepancy for anhedonia (*r* = −.56). Older participants had more discrepancy in anhedonia recall (*r* = .48). Higher income was associated with improved recall of negative self-thoughts (*r* = −.64) and trouble sleeping (*r* = −.59) and higher education was associated with better recall of negative self-thoughts (*r* = −.60).

Day 14 pain intensity was associated with discrepancies in recalled pain (*r* = −.52, *p* =.029) and appetite (*r* = −.480, *p* =.044). Further, discrepancies in trouble concentrating recall were associated with depressed mood and negative self-thoughts on day 14 (*r* = −.67, *p* =.002 and *p* = −.72, *p* <.001, respectively). No significant associations were found for day 14 anhedonia, trouble sleeping, fatigue, appetite, and trouble concentrating with recall discrepancy.

## Discussion

We found preliminary evidence of recall bias in symptom reporting for pain intensity and depressive symptoms in OAMM. While small on average, some of the discrepancies had medium effect sizes, such as for pain intensity – with a difference equivalent to 3.3 points on the 0–10 pain intensity visual analogue scale. This level of discrepancy has real-world implications. Using the World Health Organization analgesic ladder as a guide, a patient who reports a pain score of 7 (severe pain) in the office could well receive a prescription for an opioid medication. In contrast, if the same patient reported a pain intensity score of 4 (moderate pain) via EMA symptom monitoring, they would likely be prescribed a non-opioid medication to mitigate pain.

Our findings support a small but expanding literature on recall bias for pain and depressive symptoms – albeit with younger populations – including overreporting at recall in knee osteoarthritis (Previtali *et al*., 2022) and functional bowel syndromes (Jones *et al*., 2022). While we found that some baseline characteristics such as pain intensity were associated with greater discrepancies, no clear patterns emerged in line with prior research (Jones *et al*., 2022). Similar to our findings of recency effects, a study on a non-clinical sample of younger adults found evidence of peak end bias, with more recent symptoms being more highly correlated than average scores over a two-week period for the PHQ-9 (Horwitz *et al.*, 2023). Further research into recall bias in OAMM is needed, including identifying predictors of recall discrepancy.

To our knowledge, this is the first study of recall bias for pain and depression symptoms in OAMM, with additional strengths being the use of EMA to prospectively measure daily symptoms, and the wide age range of our sample (67–95). However, several limitations should be noted, with the main limitation being the discrepancy between the EMA items range and the follow-up questionnaires, which we handled via transformations. While these findings should be considered preliminary due to this discrepancy and there is a need for additional studies using consistent ranges, two items should be highlighted despite the transformations. First, if the cut-offs we used for EMA item ranges were incorrect, we would expect consistent bias across all variables; yet while some symptoms were overreported (e.g., pain and depressed mood), other symptoms had almost no difference (e.g., negative thoughts of self), and we found no correlations between others (e.g., prospective and retrospective reports of appetite and trouble concentrating). Further, while transformations can affect some analyses, correlations are not as impacted by scoring, and even with a different transformation we would expect the correlation patterns to be the same. Thus, although interpretation would be simpler if transformations were not necessary, our current results cannot be explained by the need for transformations. Finally, we did not assess baseline cognitive function beyond excluding participants with chart-documented or self-reported dementia diagnoses and, relatedly, depressive symptoms may have impacted memory (Singh-Manoux *et al*., 2010; Nafilyan *et al*., 2025). Considering mild cognitive impairment may be as high as 50–60% in primary care settings (Kulshreshtha *et al*., 2024; Summanwar *et al*., 2025) where it often goes unrecognized (Liu *et al*., 2024), additional studies are needed that can determine differential rates of recall bias based on cognitive function. Given our sample composition, it is also possible that OAMM without depressive symptoms would display less recall bias in pain symptoms. However, given the high co-occurrence of chronic pain and depressive symptoms in older adults (Bair *et al*., 2003; Mcqueenie et al., 2021), these findings are still relevant to this population.

Recall bias is a significant issue for clinical research, and an even bigger one for primary care providers. Chronic pain and depression care are typically delivered in primary care, where there is limited time and competing priorities when caring for OAMM. Even when structured assessments are used, the most common questionnaires for both pain (e.g., Brief Pain Inventory, Cleeland and Ryan, 1994, PEG Scale Assessing Pain Intensity and Interference, Krebs *et al*., 2009, PROMIS Pain intensity Scale, Cella *et al.*, 2010), and depression (PHQ-9, Kroenke *et al.*, 2001, Geriatric Depression Scale, Sheikh and Yesavage, 1986) rely on retrospective assessments covering periods between one and two weeks. Notably, while we found evidence of recall bias in a two-week period, primary care visits may happen months apart, and OAMM are asked to recall long periods. Better quality data on pain intensity and depression symptoms are needed for OAMM. EMA-based approaches to remote symptom assessment in the inter-visit period, such as automated tools or daily dairies, may offer patients and providers better quality data to aid decision making.

## Conclusions

Our sample size precludes us from generalizing these findings, and the use of a different response scale for EMA and recall items limits some of our findings. However, even with correlations which are not affected by scoring transformation, we saw notable patterns. Given the reliance on retrospective assessment in primary care settings for OAMM, these provocative data need to be confirmed in larger studies to establish the magnitude of symptom recall discrepancies in larger samples of OAMM, as well as mitigating strategies to better care for this population in primary care.

## Data Availability

The study data set cannot be publicly shared as consent was not sought from participants to allow for the publication of deidentified data sets. Analytic code is available upon request.
